# Unintended Consequences of Data Sharing Under the Meaningful Use Program

**DOI:** 10.2196/52675

**Published:** 2024-11-14

**Authors:** Irmgard Ursula Willcockson, Ignacio Herman Valdes

**Affiliations:** 1 Astronaut, LLC, 7505 Fannin Street, Suite 170, Houston, TX, 77054, United States, 1 7132492874

**Keywords:** electronic health records, EHR, medical record, interoperability, health information interoperability, clinical burden, Medicare, Medicaid, reimbursement, data science, data governance, data breach, cybersecurity, privacy

## Abstract

Interoperability has been designed to improve the quality and efficiency of health care. It allows the Centers for Medicare and Medicaid Services to collect data on quality measures as a part of the Meaningful Use program. Covered providers who fail to provide data have lower rates of reimbursement. Unintended consequences also arise at each step of the data collection process: (1) providers are not reimbursed for the extra time required to generate data; (2) patients do not have control over when and how their data are provided to or used by the government; and (3) large datasets increase the chances of an accidental data breach or intentional hacker attack. After detailing the issues, we describe several solutions, including an appropriate data use review board, which is designed to oversee certain aspects of the process and ensure accountability and transparency.

## Introduction

### Background

Interoperability has been an overarching goal of the American health care industry since the American Recovery and Reinvestment Act in 2009. Provider-to-provider sharing of patient information was designed to improve the safety, quality, and efficiency of patient care. The next target for interoperability was provider-to-patient sharing of information. The 21st Century Cures Act called for patients to have electronic access to their health care record [1]. Health Level 7 Fast Healthcare Interoperability Resources was developed for secure data exchange between computer systems using different information storage methods [2].

Interoperability is a prerequisite for meaningful use (MU). The Health Information Technology for Economic and Clinical Health Act, a component of the American Recovery and Reinvestment Act, included incentives for providers to adopt electronic health records (EHRs) as well as penalties for failing MU [3]. The Meaningful Use program requires providers to share data with the Centers for Medicare and Medicaid Services (CMS) to ensure satisfactory health care quality. Using the Quality Reporting Document Architecture [4], providers are required to submit data in support of the quality measures applicable to their practice. If covered providers do not submit data, their rate of reimbursement is reduced.

Although interoperability has been designed to improve the quality and efficiency of patient care, there are unintended consequences of data sharing. To date, few articles have examined the negative consequences of data sharing by providers to governmental entities [5,6]. Data must be generated before it can be shared, so this article begins with clinical data generation.

### Ethical Considerations

This study does not include human subject research (no human subject experimentation or intervention was conducted) and so does not require institutional review board (IRB) approval.

## Data Generation

Data are generated by both providers and patients during a visit. While most data are generated to serve clinical needs, some are generated solely to satisfy later MU reporting.

### Uniform Data Generation

The criteria for satisfying a particular MU requirement do not consider differences in specialty. For example, annual screening for depression is one MU measure (Centers for Medicare and Medicaid Services 2, version 12, 2023) [7]. Patients with depression are frequently undiagnosed in general medicine settings and therefore remain untreated. Explicit screening for depression in these settings is important to identify patients and offer appropriate treatment [8,9]. Administering a validated screening instrument is one way to satisfy the screening portion of the MU requirement.

In contrast, psychiatrists screen patients for depression using implicit methods such as interviewing the patient [10,11]. Requiring them to report an instrument score for each patient does not further the goal of identifying and treating patients with depression. It does, however, increase the burden of data collection and documentation on both the provider and patient.

### Proposed Solution: Broader Criteria for Meeting MU Requirements

Instead of forcing all providers to perform the same task to meet a particular MU requirement, providers should be allowed limited flexibility to decide how best to meet the intent of the requirement.

### Free-Rider Problem

In the broadest definition, a free rider receives a benefit without contributing to the cost of that benefit’s production [12]. Billing codes determine the provider’s compensation for a given patient visit. These codes in part represent the amount of time spent on a given activity. However, there is no billing code for collecting data only required for CMS reporting. Providers are penalized for not providing the data but are not compensated for their time spent; thus, the CMS is acting as a free rider.

### Proposed Solution: Current Procedural Terminology Code Modifiers

Current Procedural Terminology code modifiers further describe a procedure code without changing its definition [13]. Creating a modifier specifically for MU data collection would allow providers to bill for the time spent on clinical data collection. Additional payment for data collection should be made by the CMS, since it receives the reports.

## Data Ownership

### Access to Data

Ownership of data has philosophical implications that differ from ownership of real property [14,15]. A more useful framework for understanding the consequences of MU data collection may be access to data. Patients have a compelling interest in managing their own data. Patients who do not trust their provider with safeguarding their data may withhold information, leading to adverse outcomes [6]. This is especially true for marginalized groups [16].

Despite advances in interoperability and data-sharing mandates [1], neither providers nor patients can usually access all data pertaining to their role. Health care entities create data silos and deny access to patients and providers who are not, or are no longer, part of the entity. Data do not commonly follow patients who are seen by multiple providers. Outside of closed systems such as the Veterans Affairs, in which patients receive all care within the system, data do not follow providers who work in multiple health care settings, or who change jobs.

### Proposed Solution: Improved Interoperability Processes

While interoperability is constantly improving, the process of data sharing continues to be cumbersome. We propose the following process: When a patient begins receiving care at a particular entity, the consent form should include data sharing with providers outside of the entity. If the patient does not opt out, any provider who has the patient’s demographics and certifies that they need access to protected health information (PHI) should receive it. Allowing patients to consent ahead of time lowers the burden on both the patients and the providers.

## Data Sharing

### Overview

Data can be shared with the CMS either by a provider via EHRs or by an insurance company. The CMS receives complete charts, including all PHI. Neither providers nor patients are directly involved in this process, and they may not know when or how often data sharing occurs.

### Calculating Compliance

Many MU measures include a numerical criterion. To calculate the percentage of patients who have had a particular test or screening requires collecting not only the charts of patients satisfying the measure (the numerator) but also the charts of patients not satisfying the measure (the denominator). In practice, this means that any chart may be included in any dataset, and that many charts are included in multiple datasets. Patients and providers have no control over this process and no way of knowing which charts are included in which dataset.

### Lack of Patient Consent

Patient consent forms include consent for sharing data with other members of their health care team, designated family members, and insurance companies. Currently, consent forms do not include sharing data with the CMS as part of MU requirements. Patients are unaware that their data are shared with the CMS, via their providers and their insurance companies, and have no mechanism to give or deny consent.

### Proposed Solution: Updated Consent Forms and Opt Outs

Including a section on data sharing with the CMS in patient consent forms is straightforward. Both providers and insurance companies can include a section explaining that PHI may be shared with the CMS and allow patients to opt out.

However, opting out could have unintended consequences. Some patients may hesitate to share any health data with the CMS. More patients may hesitate to share sensitive data, such as mental health or substance misuse data, with the CMS. Therefore, the CMS would need to decide how to handle compliance calculations. Giving patients the opportunity to opt out of data sharing also requires changes to EHR programming.

### Data Use and Potential Misuse

The intent of MU data collection is to improve the efficiency and effectiveness of health care. However, once collected, data can be used for any number of other purposes. At present, it is unclear what safeguards are in place to prevent other branches of government from accessing the data for their own purposes. For example, California allows a person to obtain a driver’s license without proof of legal immigration status. The personal data collected can then be accessed by the Department of Homeland Security to perform civil immigration enforcement [17].

### Data Breaches

The US government has experienced multiple data breaches over the last 15 years, both accidental and through hacking [18-21]. Improvements in processes at least partially address accidental breaches. Hacking by sophisticated foreign entities is more difficult to prevent. Hackers may be attracted by the combination of the volume of data and its potential sensitivity [22,23].

### Proposed Solution: An Appropriate Data Use Review Board

We propose the creation of an independent board, modeled after an IRB, to address many of the concerns related to data collection and use. Research on human subjects requires the approval of an IRB [24,25]. This requirement was put in place in the United States after a series of egregiously unethical experiments was conducted. Each IRB is required to have at least 5 members, with at least one whose main concern is scientific, one whose main concern is nonscientific, and one who is not affiliated with the academic institution in which the proposed research would take place.

As the data recipient is the CMS, a part of the federal government, we propose the following composition for an appropriate data use review board, made up of a minimum of 6 members to allow for group decision-making:

- At least one member who is employed at the CMS
- At least one member who is a clinician providing data
- At least one member who represents patients
- At least one member with communication experience
- At least one member who is a biomedical informatician with big data expertise
- At least one member who owns and operates a small market-share EHR software company

Each of the members of the appropriate data use review board has a critical role to play. As the data recipient, the CMS needs to lay out both the data required and the rationale behind collecting the data. The clinician and EHR owner provide important insight into the impact of data collection on workflows, as well as the feasibility of modifying software to streamline data collection and reporting. The patient representative provides the patients’ perspective as well as communication guidance. As any decision reached by the review board needs to be communicated clearly and effectively to patients, the communication specialist and patient representative would work together to craft and disseminate necessary information. The biomedical informatician can assist the CMS with deciding on data needs as well as suggest the most current data analysis methods. They can also help the patient representative and communication specialist explain different ways to protect patient data from unauthorized disclosure.

After an open application and vetting process, members to the board should be appointed by a bipartisan committee of the US House. Their term of service should be 4-5 years to allow members to become proficient in their roles.

Proposed uses of data should be approved by the committee and communicated to the public. Public input should be sought through a variety of means and become an important aspect of decision-making. Disclosure of data use and data breaches should be prompt and effective, without further compromising data security.

### Effect of MU on the EHR Ecosystem

MU requirements affect not only patients and providers but also EHR companies. MU certification and recertification is time consuming and costly. While EHR companies with a large market share can justify the expense and pass the cost on to their customers, companies with a smaller market share cannot. Lack of certification leads to decreased market share, thereby encouraging consolidation across the industry.

Consolidation may be advantageous to the government because it is easier to negotiate with fewer companies. However, consolidation leads to increased costs for providers and less competition. It makes independent, autonomous practice, away from corporate monocultures, very difficult. Customer service also suffers because customers have fewer choices.

Customer service is not the only casualty; innovation is also affected. Smaller companies are more likely to produce innovative products. However, given the high bar of MU certification, bringing these innovations to the market often proves to be cost prohibitive. Similarly, though open-source software has also driven down costs and spurred innovation [26], given the expense associated with MU certification, many companies committed to open-source software have stopped providing their code freely.

## Conclusion

MU was made possible by progressive advances in interoperability. While CMS data collection has the potential to advance health care, it leads to the aggregation of large datasets that are vulnerable to unintentional data breaches and data misuse. Since the data collection is largely invisible to both providers and patients, an appropriate data use review board is needed to protect all participants.

## Abbreviations

CMS: Centers for Medicare and Medicaid Services
EHR: electronic health record
IRB: institutional review board
MU: meaningful use
PHI: protected health information
